# How Worried Are You about Food Fraud? A Preliminary Multi-Country Study among Consumers in Selected Sub-Saharan African Countries

**DOI:** 10.3390/foods12193627

**Published:** 2023-09-29

**Authors:** Jan Mei Soon-Sinclair, Samuel Imathiu, Adewale Olusegun Obadina, Fabrice Fabien Dongho Dongmo, Alex Dimitri Tchuenchieu Kamgain, Ennet Moholisa, Courage Kosi Setsoafia Saba, Abel Wilson Walekhwa, Henry Hunga, Jamal Kussaga

**Affiliations:** 1 Faculty of Allied-Health and Wellbeing, University of Central Lancashire, Preston PRI 2HE, UK; 2 Department of Food Science and Technology, Jomo Kenyatta University of Agriculture and Technology, Nairobi 00200, Kenya; samuel.imathiu@jkuat.ac.ke; 3 Department of Food Science and Technology, Federal University of Agriculture, Abeokuta P.M.B 2240, Nigeria; obadinaw@gmail.com; 4 Department of Biotechnology and Food Technology, University of Johannesburg, Johannesburg 2092, South Africa; 5 Department of Biochemistry, University of Douala-Cameroon, Douala 24157, Cameroon; donghofabrice@gmail.com; 6 Institute of Medical Research and Medicinal Plants Studies, Yaoundé 13033, Cameroon; kamgaina@uj.ac.za; 7 Food Evolution Research Laboratory, School of Tourism and Hospitality, University of Johannesburg, Johannesburg 2092, South Africa; 8 Agricultural Research Council-Animal Production Institute, Irene 0062, South Africa; moholisae1@arc.agric.za; 9 Department of Microbiology, Faculty of Biosciences, University for Development Studies, Tamale P.O. Box TL 1882, Ghana; csetsoafia@uds.edu.gh; 10 Infectious Diseases Epidemiology and Modelling Unit, Makerere University, Kampala P.O. Box 22418, Uganda; aww36@cam.ac.uk; 11 Department of Land Resources Conservation, Ministry of Agriculture, Lilongwe P.O. Box 30291, Malawi; henryhunga@gmail.com; 12 Department of Food Science and Agroprocessing, School of Engineering and Technology, College of Agriculture, Sokoine University of Agriculture, Morogoro P.O. Box 3000, Tanzania; kussaga@sua.ac.tz

**Keywords:** adulteration, food safety, staple foods, sub-Saharan Africa, trust, vulnerable foods

## Abstract

Food fraud is an old, recurring, and global threat to public health. It poses a serious threat to food security in sub-Saharan Africa (SSA). Despite the prevalence of food fraud in SSA, little is known about how food fraud is viewed by consumers. This study aims to provide an overview of consumers’ concerns about food fraud in SSA. A multi-country survey was conducted in October 2022–31 January 2023, and 838 valid responses were returned. To reduce the large and correlated dataset, Principal Component Analysis (PCA) was used. Five components were derived from PCA: (i) Staple foods; (ii) Premium food and drink products; (iii) Trust in reliable sources; (iv) Trust in less reliable sources; and (v) Trust in food vendors. The findings revealed Ghanaian (mean rank = 509.47) and Nigerian (mean rank = 454.82) consumers tended to score higher on the measure of food fraud concern suggesting that they were less confident in the safety and quality of the food they consume. Demographic characteristics including age, number of children, personal and family experience of food fraud and PCA components such as ‘Staple foods’, ‘Trust in reliable sources’, and ‘Trust in food vendors’ significantly predicted the model. This is the first preliminary study to provide empirical findings on consumers’ concerns about food fraud in SSA. Practical and policy recommendations for the region are suggested. This includes (i) modelling the AfriFoodinTegrity in West Africa across other major regions such as Central, East, and Southern Africa; (ii) establish a regional sub-Saharan Africa Rapid Alert System for Food and Feed (SSA-RASFF) platform; and (iii) food safety and food fraud reports could be incorporated into SSA-RASFF portal for information sharing.

## 1. Introduction

Sub-Saharan Africa (SSA) is made up of 48 countries and composed of low, lower-middle, upper-middle, and high-income countries [1]. Agriculture is one of the most important sectors of the economy. Twenty-three percent of SSA’s Gross Domestic Product (GDP) comes from agriculture [2] and major agricultural exports include cocoa, cassava, coffee, tea, and horticultural products such as fruits, vegetables, and pulses [3]. Despite exporting 4.7% (46 million tonnes out of 989 million tonnes of all crop production) of its agricultural products, there is heavy reliance on food imports. Africa imports up to US$35 billion worth of food, which is projected to reach US$110 billion by 2030 [4] to support its population. Sub-Saharan Africa has a population of 1.18 billion (2021 estimate), and this is expected to reach more than 2 billion in 2050 [5,6]. At least 123 million people (12%) of the SSA population faced acute food insecurity in 2022 [7]. This was exacerbated by COVID-19 and the war in Ukraine due to disrupted food supply chains and increased prices of agricultural inputs and food [7]. Food insecurity results in the reduction in diet quality and would push consumers away from nutritious and safe food products towards calorie-dense and potentially counterfeit food products.

Based on [8] and various local news reports, food fraud is rife in SSA (Table 1). Food fraud is the intentional modification of food products for economic gain [9]. According to the Confederation of Tanzania Industries, at least 50% of all goods, including food imported into the country were fake [10]. The drivers of food fraud are similar globally, for example, market competition, complex supply chain, inadequate governance, lack of trust and transparency, low probability of detection, quick rewards but small penalties, resource scarcity and climate change-induced weather changes [11]. However, food fraud in SSA is further compounded by conflict and security challenges [12], weak economic growth, high population growth [13], and bureaucratic corruption [14] that further disrupt the food supply chain. The combination of these factors makes SSA a perfect storm for food fraud to proliferate. 

A variety of fraudulent activities were documented in local media as shown in Table 1. This includes the production of counterfeit alcoholic beverages, honey, and seeds, adulterating palm oil with Sudan IV, artificial enhancement of animal protein, illegal trading, illegal fishing and poaching of seafood, and smuggling. Smuggling of contraband rice and vegetable oil were especially prevalent in Nigeria [15,23]. Food fraud threatens the economy and could potentially lead to public health threats due to food safety issues [24]. In fact, food fraud compromises most of the UN Sustainable Development Goals by reducing the availability of safe and quality food products, destabilises food security, weakens food sustainability, crowds out legitimate economic activity, undermines private sector contributions to economic growth and employment, and deprives government of tax revenues for investments in public services. However, there is scant research in this area of concern and most reports were captured from local news or through the food control authorities. It is also unclear whether consumers from different countries view food fraud differently and what their level of concern is. An understanding of consumers’ perception and concerns about food fraud would assist responsible producers and manufactures’ decision in technology investment to address food frauds. Our findings can help policymakers and researchers to identify key areas of concern that could be targeted in efforts to improve food safety, quality and public health nationally, regionally and globally. Government authorities in SSA can also use the study results to design and implement policies that aim at addressing food fraud and restoring consumers’ trust in food. To address these gaps, this study aimed to provide an overview of consumers’ concern(s) about food fraud in sub-Saharan Africa. 

## 2. Materials and Methods

### 2.1. Questionnaire Development

This was a multi-country, cross-sectional quantitative study. A semi-structured questionnaire was developed based on food fraud [25] and food safety studies [26,27]. The questionnaire was divided into five sections, i.e., (i) demographics; (ii) food and drinks associated with food fraud; (iii) attitudes towards food fraud; (iv) trust in government and food industry; and (v) trust in information sources. In the demographics section, participants were asked to provide information about their country of residence, gender, age, education level, residence area (e.g., urban, sub-urban, rural), number of children, frequency of food shopping and meal preparation, and if they’ve experienced food fraud. A question on ‘How do you feel about food fraud?’ was included in this section. 

Section (ii) asked participants’ views on ‘Which foods or drinks are commonly associated with food fraud in your country?’ and ‘Which supply chain do you think is commonly affected by food fraud?’. Sixteen food and drink categories including ‘alcoholic and fermented beverages’, ‘non-alcoholic beverages’, ‘cereals (e.g., maize, sorghum, millet), ‘eggs and egg products’, ‘fruits and vegetables’, ‘fats and oils’, ‘fish and fish products’, ‘herbs, spices and seasonings’, ‘milk and dairy products’, ‘meat and meat products’, ‘nuts and seeds’, ‘poultry and poultry products’, ‘prepared or ready-to-eat meals’, ‘roots and tubers’, ‘seafood (excluding fish)’, and ‘others’ were provided for participants to select. In the second question, different sectors of the food supply chain (i.e., farm, food manufacturer, distributor, wholesaler, supermarkets, small food businesses, restaurants, street food vendors, market stalls, and others) were included in the options. 

Section (iii) was measured using a Likert scale of 1–5 (where 1 = Not worried at all/Not vulnerable at all and 5 = Very worried/Very vulnerable). Specific questions on whether participants were worried about specific food fraud issues such as (a) adulteration (e.g., adding banned colourings to spices); (b) addition (e.g., adding stones to salt); (c) artificial enhancement (e.g., using formaldehyde to preserve fish); (d) dilution (diluting raw milk with water; (e) diversion (e.g., redirecting food aid to markets where aid is not required); (f) counterfeit (e.g., producing fake alcoholic drinks); (g) dilution (e.g., diluting premium vegetable oil with cheap vegetable oils); (h) mislabelling (e.g., changing expiry date and selling expired food products); (i) Smuggling (e.g., smuggling foods and drinks across borders); (j) Substitution (e.g., substituting meat with other meat species); (k) Misrepresentation (e.g., selling food or drinks below declared weight) were provided with ‘Yes’, ‘No’, and ‘Unsure’ options. Examples of food fraud incidents were given in each option to avoid ambiguity. A question on ‘How vulnerable are the following food or drink products to food fraud?’ was also posed to the participants. The food and drink categories are similar to the list provided in Section (ii).

Sections (iv) and (v) were measured using Likert scale of 1–5 where 1 = Strongly disagree and 5 = Strongly agree. Section (iv) measured participants’ trust in the government and food industry. For example, ‘I trust the following:’ (a) government is the most competent to protect public from counterfeit foods; (b) food industry is the most competent to protect public from counterfeit foods; (c) food businesses will not knowingly sell fraudulent food products; (d) small food operators will not knowingly sell fraudulent food products; (e) food regulatory agencies will take action against sellers that sell fraudulent food; (f) consumers will not buy from markets that sell fraudulent food. The final section measures consumers’ trust in information sources, such as those shared by (a) family or friends; (b) government websites; (c) World Health Organization; (d) news; (e) scientists; (f) social media; (g) magazines. 

The questionnaire was translated into Swahili and French and back-translated into English. All questionnaires were reviewed by food safety experts from participating SSA countries for relevance, content, and face validity. The study was conducted in accordance with the Declaration of Helsinki, and approved by the HEALTH Ethics Committee of University of Central Lancashire (Ref No. 0227, 13 October 2021).

### 2.2. Pilot-Testing and Data Collection

The questionnaire was pilot tested among 20 respondents located in Ghana and Malawi during September 2022. Reliability analyses were 0.973 (vulnerability of food and drink sectors), 0.674 (trust in government and food industry), and 0.872 (trust in information sources). All Cronbach’s alpha were higher than 0.60 indicating acceptable to high reliability [28]. One item from ‘Trust in government and food industry’ scale was removed. A question on ‘dilution of vegetable oil’ was also removed to avoid duplication and a question on ‘underweight product’ was modified to ‘food and drinks sold below declared weight’ to improve clarity. The questionnaire was adapted onto onlinesurvey.ac.uk platform and pre-tested to ensure the logic and sequence of questions were correct. Participants were only able to answer the survey once as the platform was set to prevent multiple entries or participation from the same respondent. Snowball, non-probabilistic sampling approach was used to invite participants. Participants were encouraged to share the online survey widely to increase number of participations. The study aimed to recruit 385 participants from each participating country based on 95% confidence level, 5% margin of error and 50% population proportion. Participants were approached through different social media platforms (e.g., Facebook, LinkedIn, and Twitter (currently known as X)) and emails. All participants were provided with an explanation of the study and consent was obtained prior to completing the survey. 

### 2.3. Statistical Analysis

Descriptive statistics, principal component analysis (PCA) and ordered logistic regression were conducted using SPSS version 28.0 (IBM, Chicago, IL, USA). Due to the large amount of data and variables, it is necessary to use a multivariate tool such as PCA. PCA helps to reduce the large dataset into fewer uncorrelated components while ensuring minimal loss of information [28]. A Kruskal–Wallis H test with Dunn’s pairwise tests was carried out to determine if concerns about food fraud were significantly different between countries. PCA was performed using varimax rotation on 27 variables which measured ‘Vulnerability of food and drink sectors’ (15 variables, please see Section iv) and ‘Trust in government and food industry’ (12 variables, please see Section v). Components with eigen value of more than 1 were retained as factors or independent variables to be used in logistic regression. Other independent variables are demographic characteristics including country, gender, age, residence, number of food fraud, self-experience of food fraud and family experience of food fraud. Food fraud concern is the dependent variable. It refers to the level of worry about food fraud. It is measured using the question ‘How do you feel about food fraud?’ and measured using Likert scale where 1 = Not worried at all to 5 = Extremely worried. Thus, ordered logistic regression is suitable as a method of analysis since it is used to analyse ordered categorical dependent variable (i.e., food fraud concern) to examine if socio-demographic factors and factors derived from the PCA significantly predict consumers’ level of worry about food fraud. A *p*-value < 0.05 was considered statistically significant. None of the pilot test data were included in the final analysis. 

## 3. Results

In total, 913 responses were received, of which 838 were valid, with no missing data. Kenya, Nigeria, and Cameroon returned the highest numbers followed by South Africa, Tanzania, and Ghana. The smaller number of participants from other SSA countries including Ethiopia, Ivory Coast, Malawi, Uganda, etc., were categorised as ‘Others’. A Kruskal–Wallis H test showed a statistically significant difference in concern about food fraud between countries, χ^2^(6) = 19.178, *p* = 0.004, where Ghana had the highest mean rank, followed by Nigeria and South Africa (Table 2). About 85.44% of participants were either worried or very worried about food fraud. More than 50% of the participants reported that they had experienced some form of food fraud. Most examples include purchasing expired food products that were re-labelled with new expiry dates, adulterated milk, adulterated palm oil, and buying misrepresented food and drink products (Table 3). 

Participants were asked if they were worried about specific food fraud issues such as adulteration (e.g., adding banned colourings to spices), counterfeit (e.g., producing fake alcoholic drinks), mislabelling (e.g., changing expiry date and selling expired foods), and other forms of fraudulent practices. Table 4 highlights the concern about different types of food fraud according to countries. Besides mislabelling and substitution, there was a statistically significant association between countries and their concern about different types of food fraud. All countries’ participants equally responded that they were concerned about mislabelling χ^2^(12) = 8.409, *p* = 0.752 and substitution χ^2^(12) = 15.511, *p* = 0.215 (Table 4).

The vulnerability of food and drink categories was significantly different according to countries (Table 5). Pairwise comparisons with adjusted *p*-values showed significant differences, especially between Cameroon and other SSA countries. For example, cereals χ^2^(6) = 34.064, *p* < 0.001 were significantly rated as less vulnerable in Cameroon compared to Kenya, Nigeria, and Tanzania. Eggs and egg products (χ^2^(6) = 19.275, *p* < 0.05) were rated as highly vulnerable in Tanzania. Pairwise comparisons showed significant differences between Tanzania with Cameroon, Kenya, and Nigeria. Meanwhile, multiple pairwise comparisons showed significant differences between countries in herbs (χ^2^(6) = 74.578, *p* < 0.001) and meat (χ^2^(6) = 39.022, *p* < 0.001) categories. Cameroon consistently rated herbs as less vulnerable compared to Ghana, Nigeria, South Africa, and Tanzania. The findings also revealed significant differences between Kenya with Ghana, Nigeria, and South Africa.

The Kaiser–Meyer–Olkin (KMO) measure verified the sampling adequacy for all analyses, KMO = 0.894 which indicates high acceptability [29]. The Bartlett’s Test of Sphericity χ^2^(351) = 8655.692, *p* < 0.001 indicating correlations between items were sufficiently large for PCA. Five components were retained based on eigenvalues of higher than 1 and explained 55.60% of the variance. Cronbach’s alpha for each component was higher than 0.60. Table 6 shows the factor loadings after rotation. Variables that cluster on the same components suggest that component 1 represents ‘Staple foods’, component 2 represents ‘Premium food and drink products’, component 3 is ‘Trust in reliable sources’, component 4 is ‘Trust in less reliable sources’, and component 5 is ‘Trust in food vendors’ behaviour’. The extracted components were checked for multicollinearity where tolerance levels were higher than 0.20 and variance inflation factor (VIF) values were below 10.00 [30]. All components were used as independent variables in the ordered logistic regression.

The likelihood ratio chi-square test [χ^2^(18) = 147.811, *p* < 0.001] indicated a significant improvement in fit compared with the null (no predictors) model. The likelihood ratio chi-square tests were significant for country, age, number of children, and self and family experience of food fraud. The PCA components such as ‘Staple foods’, ‘Trust in reliable sources’, and ‘Trust in food vendors’ behaviour’ were significant predictors (Table 7). Specific country effects were also determined in the ordered logistic regression. Ghana (OR = 2.954, *p* < 0.05) was significantly more concerned about food fraud compared to Others (‘Other countries’ is coded as the reference value). Negative values associated with South Africa and Tanzania indicated less concern about food fraud. 

Age (OR = 1.201, *p* < 0.05) and number of children (OR = 1.181, *p* < 0.05) were significant predictors of the model. As age and number of children increase, consumers were more concerned about food fraud issues. However, if consumers had experienced food fraud incidents, they were less likely to be worried about food fraud. Similarly, as trust in food vendors increases (OR = 0.835, *p* < 0.05), the model predicts a reduction in food fraud concerns. It is interesting to note that ‘Premium food & drink products’ did not significantly predict an increase in food fraud concerns. This was in contrast with ‘Staple foods’ where the independent variable significantly predicted the model (OR = 1.420, *p* < 0.001). For example, respondents were 1.24–1.63 times more likely to be worried about food fraud for each increasing unit in ‘Staple foods.’ ‘Trust in reliable sources’ (OR = 1.249, *p* < 0.05) was identified as a significant positive predictor. Consumers were 1.09–1.44 times more likely to be concerned about food fraud as trust in reliable sources increases. 

## 4. Discussion

More than half of the respondents had experienced some form of food fraud echoing the findings reported by previous studies where ripened bananas, sugar, molasses, and water were added to honey [31], milk was adulterated with water, starch and flour to increase viscosity, and non-fat total solids [32,33] and palm oil were adulterated with Sudan IV dye to appeal to customers [34]. The reported fraud incidents possibly represent the tip of the food fraud iceberg since most adulteration often remains undetected, unreported and/or uninvestigated [35]. Moreover, lack of consumers’ food safety awareness or unavailability of strong consumer organisations and inadequate food control systems could be among the reasons for the under-reporting of food fraud incidences in the SSA region. 

Consumers from Ghana demonstrated the most concern about food fraud followed closely by Nigeria. These findings were also supported by the results shown in Table 4, where consumers from Ghana were consistently worried about specific food fraud issues including adulteration, addition, artificial enhancement, and mislabelling. It is likely that Ghana consumers were more concerned about food fraud due to incidents of adulteration of palm oil using Sudan IV dye. Reports of adulteration of palm oil from West Africa had led to a ban of most palm oil sale from the region. Consumers were also alerted by the Food and Drugs Authority to purchase palm oil from reputable sources. This has led to a decline in consumers’ confidence and trust in palm oil over recent years [34,36]. Respondents from Nigeria were mostly worried about smuggling and misrepresentation of food and drink products. An example was rice smuggling which is a major concern in Nigeria. This is a result of increased rice consumption over the years and despite efforts to increase rice self-sufficiency, Nigeria remains one of the largest rice importers in the world [37]. To reduce reliance on imported rice and to improve self-sufficiency, rice tariffs and quantity controls were implemented [38]. When tax and regulatory control increases, this itself drives the informal and underground economy as the demand cannot be met through legal supply routes [39]. Rice is often smuggled into the country through the eastern and northern land borders from Benin and Niger or at ports such as Lagos [38,40]. 

The results show that as the number of children and respondents’ age increased, food fraud concern increased. This is in line with previous studies [41,42,43] where households with one or more children under 18 and older residents were more concerned about food safety, as children and older consumers are more vulnerable to food contaminations. It is interesting to note that consumers who had experienced food fraud acts personally were less likely to be worried about food fraud. This contradicts [44] where risk perception increases after a previous experience with food safety incidents. This might be due to increased awareness of food fraud; thus, consumers became more defensive and took precautionary measures to protect themselves against food fraud. Study participants were motivated to protect themselves and their family members. For example, one of the participants elaborated their coping strategy against food fraud as shown below.

*“The meat is also added with illegal preservatives so that it can have a longer shelf life. This made me quit meat consumption unless it has been slaughtered in my presence”*

(Male, Kenya).

However, the possibility that consumers are becoming ‘used to’ food fraud practices, especially if the fraud is common practice, cannot be ruled out. In this case, it is no longer a concern, but a normal practice. For example, purchasing adulterated cereal such as rice and sorghum with stones [45], buying premium rice that had been substituted with local varieties [46], and purchasing maize with remainder of cobs left in [45] are becoming normal practices. Thus, consumers who had experienced food fraud acts may be used to the practices and would utilise home-based practices such as washing and sifting through rice to remove stones, freezing cowpea to kill weevils, and washing green leafy vegetables with salt [45]. 

As trust in food vendors increases, food fraud concern decreases. These findings are comparable to previous studies where trust with specific vendors were key to reducing food safety risks. This is especially relevant for foods where safety and quality are difficult to judge. In [45], Nigerian consumers who were worried about the presence of stones in local rice prefer to buy ‘stone-free’ rice from specific, trusted vendors. In Ghana and Rwanda, trust in vendors was important when purchasing food or choosing a safe place to eat [47,48]. In general, as the rate of food fraud increases, trust in vendors and retailers plays a significant role when it comes to decision-making on product purchases.

‘Premium food and drink products’ such as animal protein, alcoholic beverages, oils, and prepared food products were considered as highly vulnerable foods. Such products are more likely to be processed or due to the physical state of the food (e.g., in liquid or minced form) which makes the food more vulnerable. However, such foods did not increase consumers’ level of concern compared to staple foods (e.g., roots, tubers, cereals, and vegetables). It is likely that premium foods were more likely to be inspected and risk reducing strategies were practiced by consumers [45]. On the other hand, consumers were increasingly worried about the status quo of staple food products and presence of adulterants in such foods. Cereals such as maize, millet, and sorghum; roots and tubers such as yams and cassava; and fruits such as plantains are essential staples in SSA. Fish provides 22% of the protein intake in SSA and are often purchased smoked or dried [49,50]. There were multiple reports of fraud associated with staple food products such as artificial enhancement of *fufu* and *garri* from cassava using chlorine bleach to whiten the products [51], using formaldehyde to preserve fish [52] and exceeding the approved doses of plant hormones to ripen plantains [53]. Studies had reported high levels of mycotoxins in cereal products, especially foods consumed by infants and young children [54,55]. Mycotoxin such as aflatoxin B_1_ in *Tom bran* (a cereal-legume weaning food) was 53 times higher than the EU threshold of 0.1 µg/kg set for baby food [56]. The deliberate sale of contaminated cereals and nuts with no controlled levels of contaminants is considered a fraudulent action [57].

An increase in ‘Trust in reliable sources’ such as reports from World Health Organization, scientists and government was more likely to increase food fraud concern. Nordhagen et al. [45] revealed that consumers placed responsibility on government to ensure food safety. Reliable sources would vet and/or investigate the incidents to ascertain the authenticity of the complaints or reports. Positive effects of trust have been observed in other studies where consumers value government certification [58,59] and scientists were rated as the most trusted source of scientific information [60,61].

### 4.1. Limitations

The study has several limitations. The number of respondents was small and not representative of the region. The sampling was based on snowball sampling approach. It was also based on self-reported experiences and perceptions of food fraud. In addition, the participants were mostly educated to tertiary level and most reside in urban areas and have greater access to media and information related to food fraud topics. This has introduced selection bias to the study. Thus, the findings should not be generalised to specific countries or SSA. However, this preliminary study has shown a snapshot of the perceptions of SSA consumers on food fraud and reflected the significance of tackling this issue. Elliott [62] reported that food issues in Africa is not only about a lack of food, but food contamination and fraud are major concerns. 

### 4.2. Implications for Research and Policy

Food fraud in SSA further compounds food insecurity in the region. Currently, there are limited food fraud studies from SSA. It is essential that more research and data are collected from the region and the rest of the world. As reported by [62], concerted effort is needed from national authorities, governments across the world, research institutions, World Bank and United Nations to tackle food fraud. One such example is the collaborative efforts between Institute for Global Food Security, Queen’s University, Belfast, and University of Cape Coast, Ghana, in establishing the Africa Centre for Food Fraud and Safety (AfriFoodinTegrity) which conducts food authenticity tests and capacity building [63]. AfriFoodinTegrity in West Africa could be modelled across other major regions including Central, East, and Southern Africa. This study indicates that there is a high level of trust in reliable sources such as those from government websites, World Health Organization, and scientists. There is a possibility for national and regional authorities to establish a regional sub-Saharan Africa Rapid Alert System for Food and Feed (SSA-RASFF) platform that enables member states to notify and exchange information on risks associated with food and feed in the region. A number of food fraud reports from SSA are currently reported within the European Commission Knowledge Centre for Food Fraud and Quality Monthly Food Fraud Summary. Similarly, food safety and food fraud reports could be incorporated into SSA-RASFF portal for information sharing and identification of issues flagged in the system. 

## 5. Conclusions

This is the first preliminary study to provide an overview of consumers’ concern about food fraud issues in SSA. Food fraud concern manifests differently for each country. Findings from this study revealed Ghana participants tended to score higher on the measure of food fraud concern suggesting that they were less confident about the food safety and quality of food they consume. Other demographic characters including age, number of children, personal and family’s experience of food fraud, and PCA components such as ‘Staple foods’, ‘Trust in reliable sources’, and ‘Trust in food vendors’ have significant impact on their level of concern in food fraud. Food fraud is a concerning but often an overlooked issue in SSA as the region struggles with food security issues. However, food fraud would further exacerbate the accessibility and availability of safe and nutritious food. It is highly recommended that more studies should be conducted in the region.

## Figures and Tables

**Table 1:** Recent food fraud examples in sub-Saharan African countries.

Country	Type of Food Fraud	Food or Drink Categories	Description	References
Burkina Faso	Illegal export	Cereals and nuts	Seized up to 600 tonnes of grains, maize, and nuts for illegal export	[15]
Burundi	Illegal trade	Dried food	Illegal trade of parchment coffee	[15]
Cameroon	Counterfeit	Honey	Production of fake honey using boiled water and sugar products	[15]
Ghana	Adulteration	Oil	Palm oils were found to be adulterated with Sudan IV dye	[15]
Ivory Coast	Smuggling	Fruits	35 tonnes of contraband cacao were seized from Ghana	[15]
Kenya	Counterfeit	Alcoholic beverages	More than 300 cartons of counterfeit spirits were confiscated	[16]
Mozambique	Illegal fishing, misrepresentation of origin, and falsified documents	Seafood	Four tonnes of fish were illegally caught	[17]
Namibia	Counterfeit	Alcoholic beverages	Dismantled 120 illegal distilleries and seized over 6000 litres of smuggled liquor	[15]
Nigeria	Smuggling	Cereal	Over 90,000 bags of 50 kg rice were seized	[18]
Rwanda	Adulteration	Honey	Honey adulterated with sugar syrup and crushed yellow bananas	[19]
South Africa	Illicit trade	Seafood	Poaching and illicit abalone trade to East Asia	[20,21]
Tanzania	Counterfeit	Alcoholic beverages	Production and sale of illicit alcoholic beverages	[22]
Uganda	Artificial enhancement	Poultry and meat	Chicken and pigs were fed with anti-retroviral drugs to accelerate growth	[15]
Zimbabwe	Counterfeit	Seeds	Fake maize seeds were seized	[15]

**Table 2:** Demographics characteristics of participants from sub-Saharan Africa (*n* = 838).

Items	Description	Frequency (%)
Country	Kenya	264 (31.5)
	Nigeria	190 (22.7)
	Cameroon	140 (16.7)
	South Africa	81 (9.7)
	Tanzania	60 (7.2)
	Ghana	54 (6.4)
	Others	49 (5.8)
Gender	Male	428 (51.1)
	Female	410 (48.9)
Age	18–29	304 (36.3)
	30–39	225 (26.8)
	40–49	182 (21.7)
	50–59	88 (10.5)
	60 and above	39 (4.7)
Residence	Urban	622 (74.2)
	Sub-urban	171 (20.4)
	Rural	45 (5.4)
Number of children	0	281 (33.5)
	1–2	298 (35.6)
	3–4	208 (24.8)
	5–6	29 (3.5)
	More than 6	22 (2.6)
Have you experienced food fraud	Yes	444 (53.0)
	No	137 (16.3)
	Unsure	257 (30.7)
Have your family members experienced food fraud	Yes	367 (43.8)
	No	133 (15.9)
	Unsure	338 (40.3)
Worried about food fraud		Mean rank
	Ghana	509.47 *
	Nigeria	454.82
	South Africa	403.86
	Cameroon	402.97
	Others	401.13
	Kenya	401.00 *
	Tanzania	382.78 *

* *p* < 0.05.

**Table 3:** Examples of personal experiences of food fraud.

Country	Respondent	Personal Experiences of Food Fraud
Cameroon	Female	I’ve bought honey which were weighed down with items such as banana
Cameroon	Female	I purchased expired products such as milk and mayonnaise that had been updated with new expiry dates
Ethiopia	Male	Butter mixed with banana and vaseline and berbere (mixed spice) mixed with clay soil
Ghana	Male	The palm oil I bought in an open market tasted different after use. Initially, the enhanced color appeals to me, it was after using the product that we realized artificial color was added.
Kenya	Male	Milk, it seems like starch and margarine had been added to it
Kenya	Female	Bought a basket of beans with stones
Kenya	Male	Rotten corn was placed under good quality corn to increase the weight
Kenya	Male	The milk vendors around my home especially and urban areas have the tendency of diluting the milk so that they can fetch a higher price.
Kenya	Male	Milk was so sticky like they have been mixed with flour or something like that, cause it wasn’t just milk.
Kenya	Male	Obambla (traditional dried fish in Kenya) was coated with food colouring to make it look fresh
Nigeria	Female	Dry pepper mixed with shaft and color to increase quantity and appearance
South Africa	Female	Rotten meat cuts were packed under fresh meat cuts, so I could only see the fresh meat through the cling wrap packaging.
Tanzania	Male	I’ve bought sorghum and millet with sand added to increase the weight
Tanzania	Female	I’ve bought bread that was coloured with yellow colouring but sold as bread with egg
Tanzania	Male	At the market where I was told that a chicken is a local breed but was confirmed at home to be a hybrid and not purely local breed.
Tanzania	Male	Rice mixed with oil to make it shiny. When you wash it, you could see oil floating on top. I also witnessed rotten corn mixed with good ones and made into flour.

**Table 4:** Concern about different types of food fraud (*n* = 838).

Type of Food Fraud	Chi-Square	Cramer’s V	Cameroon (*n* = 140)	Ghana (*n* = 54)	Kenya (*n* = 264)	Nigeria (*n* = 190)	South Africa (*n* = 81)	Tanzania (*n* = 60)	Others (*n* = 49)
Adulteration	33.580 **	0.142	87.1%	98.1%	89.8%	97.4%	88.9%	86.7%	89.8%
Addition	24.405 *	0.121	67.9%	90.7%	80.3%	78.9%	67.9%	66.7%	75.55
Artificial enhancement	55.770 **	0.182	86.4%	100%	90.9%	95.8%	72.8%	85.0%	85.7%
Counterfeit	30.424 *	0.135	92.9%	87.0%	88.3%	85.8%	76.5%	78.3%	73.5%
Dilution	35.338 **	0.145	75.0%	77.8%	87.9%	52.6%	86.4%	95.0%	84.0%
Diversion	58.367 **	0.187	60.0%	74.1%	76.5%	70.0%	59.3%	38.3%	63.3%
Mislabelling	8.409	0.071	92.9%	98.1%	93.9%	97.4%	95.1%	95.0%	93.9%
Misrepresentation	22.849 *	0.117	79.3%	83.3%	89.4%	91.6%	86.4%	88.3%	87.1%
Smuggling	35.603 **	0.146	66.4%	75.9%	66.3%	80.0%	75.3%	60.0%	59.2%
Substitution	15.511	0.096	80.0%	87.0%	89.0%	87.4%	92.6%	83.3%	87.8%

** *p* < 0.001; * *p* < 0.05.

**Table 5:** Vulnerable food and drink categories according to countries (*n* = 838).

Food and Drink Categories	Kruskal–Wallis	Cameroon	Ghana	Kenya	Nigeria	South Africa	Tanzania	Others
	(χ^2^)	Mean Rank						
Alcoholic and fermented beverages	8.075	434.26 ^a^	401.12 ^a^	443.05 ^a^	388.88 ^a^	412.21 ^a^	392.99 ^a^	433.94 ^a^
Non-alcoholic beverages	7.387	394.45 ^a^	446.34 ^a^	405.43 ^a^	438.49 ^a^	437.87 ^a^	455.23 ^a^	389.54 ^a^
Cereals (e.g., maize, sorghum, millet)	34.064 **	322.47 ^bc^	429.80 ^ac^	452.69 ^a^	441.07 ^a^	386.98 ^ac^	461.33 ^a^	425.48 ^ac^
Eggs and egg products	19.275 *	398.75 ^b^	472.20 ^a^	406.65 ^b^	394.37 ^b^	424.38 ^a^	523.08 ^a^	452.50 ^a^
Fruits and vegetables	12.786	391.78 ^a^	493.72 ^a^	416.33 ^a^	434.66 ^a^	371.18 ^a^	454.25 ^a^	412.52 ^a^
Fats and oils	26.129 **	339.71 ^b^	476.44 ^a^	426.83 ^ac^	441.65 ^ac^	403.87 ^a^	473.32 ^ac^	419.26 ^a^
Fish and fish products	8.271	384.55 ^a^	430.12 ^a^	430.94 ^a^	415.99 ^a^	423.37 ^a^	474.43 ^a^	385.99 ^a^
Herbs, spices, and seasonings	74.578 **	318.05 ^b^	540.39 ^a^	371.02 ^b^	475.32 ^a^	503.97 ^a^	463.02 ^a^	427.93 ^a^
Milk and dairy products	17.870 *	378.05 ^ac^	398.85 ^ad^	450.64 ^bd^	388.01 ^ad^	422.33 ^ad^	473.53 ^ad^	444.20 ^ad^
Meat and meat products	39.022 **	342.55 ^bc^	427.65 ^ab^	457.87 ^a^	389.84 ^b^	505.64 ^a^	423.88 ^ab^	390.91 ^ab^
Nuts and seeds	68.994 **	284.85 ^b^	498.23 ^a^	409.29 ^a^	473.91 ^a^	436.40 ^a^	453.97 ^a^	491.36 ^a^
Poultry and poultry products	37.551 **	324.25 ^bc^	427.95 ^ac^	444.51 ^a^	416.97 ^a^	504.48 ^a^	434.31 ^a^	398.81 ^ac^
Prepared or ready to eat meals	10.335	387.01 ^a^	455.71 ^a^	420.58 ^a^	406.91 ^a^	478.80 ^a^	416.97 ^a^	420.47 ^a^
Roots and tubers	21.278 *	354.94 ^bc^	443.99 ^ac^	407.28 ^ac^	471.75 ^a^	428.75 ^ac^	419.52 ^ac^	424.88 ^ac^
Seafood (Excluding fish)	16.214 *	364.49 ^bc^	437.60 ^ac^	419.24 ^ac^	431.44 ^ac^	488.12 ^a^	391.59 ^ac^	432.58 ^ac^

Values with different ^abcd^ superscripts within a row indicate significant differences among countries where ** *p* < 0.001; * *p* <0.05.

**Table 6:** Loading factors and principal component analysis of observed variables.

Components	Rotated Loading Factors	% Variance Explained	Cronbach’s Alpha
Component 1: Staple foods			
Roots and tubers	0.817	26.356	0.857
Nuts and seeds	0.768		
Eggs and egg products	0.702		
Fruits and vegetables	0.676		
Cereals	0.627		
Seafood	0.594		
Fish	0.556		
Herbs	0.516		
Component 2: Premium food and drink products			
Milk	0.750	11.858	0.851
Meat	0.722		
Non-alcoholic beverages	0.667		
Fats and oils	0.651		
Alcoholic and fermented beverages	0.634		
Prepared meals	0.622		
Poultry	0.532		
Component 3: Trust in reliable sources			
World Health Organisation	0.756	7.845	0.731
Government websites	0.733		
Government’s competence	0.644		
Scientist	0.607		
Food industry competence	0.565		
Component 4: Trust in less reliable sources			
Social media	0.820	5.556	0.730
Magazines	0.792		
News	0.696		
Family and friends	0.413		
Component 5: Trust in food vendors’ behaviour			
Food industry will not sell fraudulent products	0.873	3.983	0.671
Small food operators will not sell fraudulent products	0.864		
Consumers will not buy fraudulent products	0.530		

**Table 7:** Ordered logistic regression predicting likelihood of consumers feeling worried about food fraud.

Dependent Variables	B(SE)	Odds Ratio	95% Confidence Interval
Cameroon	0.545 (0.340)	1.725	[0.886–3.358]
Ghana	1.083 (0.423) *	2.954	[1.289–6.769]
Kenya	0.254 (0.310)	1.289	[0.701–2.368]
Nigeria	0.279 (0.321)	1.322	[0.705–2.478]
South Africa	−0.047 (0.364)	0.954	[0.467–1.948]
Tanzania	−0.426 (0.379)	0.653	[0.311–1.372]
Others	0	1	
Gender	0.108 (0.144)	1.115	[0.840–1.479]
Age	0.183 (0.073) *	1.201	[1.041–1.385]
Residence	0.070 (1.279)	1.072	[0.835–1.378]
Number of children	0.166 (0.075) *	1.181	[1.019–1.369]
Self-experience of food fraud	−0.360 (0.106) **	0.698	[0.567–0.858]
Family experience of food fraud	−0.258 (0.107) *	0.773	[0.627–0.952]
Premium food products	0.129 (0.072)	1.13	[0.989–1.309]
Staple foods	0.351 (0.070) **	1.420	[1.238–1.629]
Trust in reliable sources	0.222 (0.072) *	1.249	[1.085–1.437]
Trust in less reliable sources	0.043 (0.069)	1.044	[0.911–1.195]
Trust in food vendors’ behaviour	−0.180 (0.069) *	0.835	[0.729–0.956]

** *p* < 0.001; * *p* < 0.05.

## Data Availability

Data available on request due to restrictions, e.g., privacy or ethical.

## References

[1] World Bank The World Bank in Africa. https://www.worldbank.org/en/region/afr/overview.

[2] Goedde L., Ooko-Ombaka A., Pais G., Winning in Africa’s Agricultural Market Private-Sector Companies Can Find Practical Solutions to Enter and Grow in Africa’s Agricultural Market. https://www.mckinsey.com/industries/agriculture/our-insights/winning-in-africas-agricultural-market.

[3] Giller K.E. (2020). The food security conundrum of sub-Saharan Africa. Glob. Food Secur..

[4] UNCTAD COVID-19: A Threat to Food Security in Africa. https://unctad.org/news/covid-19-threat-food-security-africa.

[5] Bjørndal T., Lappo A., Dey M., Lem A., Child A. (2016). Economic Analysis of Food Supply and Demand in Sub-Saharan Africa up to 2022—Special Focus on Fish and Fishery Products. FAO Fisheries and Aquaculture Circular No. 1101.

[6] World Bank Population, Total—Sub-Saharan Africa. https://data.worldbank.org/indicator/SP.POP.TOTL?locations=ZG.

[7] IMF (2022). Building a more food-secure Sub-Saharan Africa. Regional Economic Outlook Analytical Note: Sub-Saharan Africa—Living on the Edge.

[8] Onyeaka H., Ukwuru M., Anumudu C., Anyogu A. (2022). Food fraud in insecure times: Challenges and opportunities for reducing food fraud in Africa. Trends Food Sci. Technol..

[9] Spink J., Moyer D.C. (2013). Understanding and combating food fraud. Food Technol..

[10] The Citizen Over Half of Imported Goods Used in Tanzania Are Fake. The Citizen. https://www.thecitizen.co.tz/News/Over-half-of-imported-goods-used-in-Tanzania-are-fake/1840340-3253536-bcm3lsz/index.html.

[11] Manning L. (2016). Food fraud: Policy and food chain. Curr. Opin. Food Sci..

[12] Anderson W., Taylor C., McDermid S., Ilboudo-Nebie E., Seager R., Schlenker W., Cottier F., De Sherbinin A., Mendeloff D., Markey K. (2021). Violent conflict exacerbated drought-related food insecurity between 2009 and 2019 in sub-Saharan Africa. Nat. Food.

[13] Wudil A.H., Usman M., Rosak-Szyrocka J., Pilar L., Boye M. (2022). Reversing years for global food security: A review of the food security situation in Sub-Sharan Africa (SSA). Int. J. Environ. Res. Public Health.

[14] Olabiyi O.M. (2021). The effect of bureaucratic corruption on household food insecurity: Evidence from Sub-Saharan Africa. Food Secur..

[15] European Commission (EC) Month Summary of Articles on Food Fraud and Adulteration. Knowledge Centre for Food Fraud and Quality. https://knowledge4policy.ec.europa.eu/food-fraud-quality/monthly-food-fraud-summary-reports_en.

[16] Securing Industry Counterfeit Spirits Seized in Kenya Operation. https://www.securingindustry.com/food-and-beverage/counterfeit-spirits-seized-in-kenya-operation/s104/a15058/#.ZAm4o3bP1PY.

[17] Club of Mozambique Mozambique: Inspection Seizes over Four Tonnes of Fish in Zambezia, 10 February. https://clubofmozambique.com/news/mozambique-inspection-seizes-over-four-tonnes-of-fish-in-zambezia-209315/.

[18] Opinion Nigeria Breaking: Customs FOU Zone A Seizes Smuggled Goods Worth N13.9bn. https://www.opinionnigeria.com/breaking-customs-fou-zone-a-seizes-smuggled-goods-worth-n13-9bn/.

[19] Kagire L.M. Surge in Adulterated Honey Puts Local Industry at Risk. The New Times, 8 March. https://www.newtimes.co.rw/article/184505/News/surge-in-adulterated-honey-puts-local-industry-at-risk.

[20] De Greef K., Raemaekers S. (2014). South Africa’s Illicit Abalone Trade: An Updated Overview and Knowledge Gap Analysis.

[21] Givetash L. How Asian Appetites for Abalone—‘White Gold’—Foster Crime in South Africa That Threatens Diners’ Safety. South China Morning Post, 28 January. https://www.scmp.com/magazines/post-magazine/long-reads/article/3208034/white-gold-how-south-africas-illegal-abalone-trade-enriches-gangs-while-threatening-safety-asian.

[22] The Citizen How Fake Liquor Is Hitting Government and Distilling Firms. The Citizen. https://www.thecitizen.co.tz/tanzania/news/business/how-fake-liquor-is-hitting-government-and-distilling-firms-3751056.

[23] Golub S.S. (2012). Entreport trade and smuggling in West Africa: Benin, Togo and Nigeria. World Econ..

[24] Manning L., Soon J.M. (2016). Food safety, food fraud and food defense: A fast evolving literature. J. Food Sci..

[25] Charlebois S., Schwab A., Henn R., Huck C.W. (2016). Food fraud: An exploratory study for measuring consumer perception towards mislabeled food products and influence on self-authentication intentions. Trends Food Sci. Technol..

[26] Bolek S. (2020). Consumer knowledge, attitudes and judgments about food safety: A consumer analysis. Trends Food Sci. Technol..

[27] Ha T.M., Shakur S., Do K.H.P. (2019). Consumer concern about food safety in Hanoi, Vietnam. Food Control.

[28] Hair J.F., Anderson R.E., Tatham R.L., Black W.C. (1995). Multivariate Data Analysis.

[29] Field A. (2018). Discovering Statistics Using IBM SPSS Statistics.

[30] Menard S. (2022). An introduction to logistic regression diagnostics. Applied Logistic Regression Analysis.

[31] Damto T. (2021). A review on status of honey adulteration and their detection techniques in Ethiopia. J. Nutr. Food Sci..

[32] Handford C.E., Campbell K., Elliott C.T. (2015). Impacts of milk fraud on food safety and nutrition with special emphasis on developing countries. Compr. Rev. Food Sci. Food Saf..

[33] Nyokabi S.M., de Boer I.J.M., Luning P.A., Korir L., Lindahl J., Bett B., Oosting S.J. (2021). Milk quality along dairy farming systems and associated value chains in Kenya: An analysis of composition, contamination and adulteration. Food Control.

[34] MacArthur R.L., Teye E., Darkwa S. (2020). Predicting adulteration of palm oil with Sudan IV dye using shortwave handheld spectroscopy and comparative analysis of models. Vib. Spectrosc..

[35] Soon J.M., Krzyzaniak S.C., Shuttlewood Z., Smith M., Jack L. (2019). Food fraud vulnerability assessment tools used in food industry. Food Control.

[36] Andoh S.S., Nuutinen T., Mingle C., Roussey M. (2019). Qualitative analysis of Sudan IV in edible palm oil. J. Eur. Opt. Soc. Rapid Publ..

[37] Obayelu O.A., Agbohin J.A., Obisesan O.O. (2022). Consumers’ preference for local rice brands in Ibadan Metropolis, Nigeria. Food Agric. Econ. Rev..

[38] Hoffmann L.K., Melly P. (2018). Incentives and constraints of informal trade between Nigeria and its neighbours. West African Papers, No. 16.

[39] Soon J.M., Manning L. (2018). Food smuggling and trafficking: The key factors of influence. Trends Food Sci. Technol..

[40] Johnson M.E., Dorosh P. (2017). Tariffs, smuggling and economic welfare: A spatial analysis of Nigerian rice policy options. J. Afr. Econ..

[41] Sugimoto T., Shinozaki R., Naruse T., Miyamoto Y. (2014). Who was concerned about radiation, food safety, and natural disasters after the Great East Japan earthquake and Fukushima catastrophe? A nationwide cross-sectional survey in 2012. PLoS ONE.

[42] Liguori J., Trubswasser U., Pradeilles R., Le Port A., Landais E., Talsma E.F., Lundy M., Béné C., Bricas N., Laar A. (2022). How do food safety concerns affect consumer behaviors and diets in low- and middle-income countries? A systematic review. Glob. Food Secur..

[43] Liu P., Ma L. (2016). Food scandals, media exposure, and citizens’ safety concerns: A multilevel analysis across Chinese cities. Food Policy.

[44] Zanetta L.A., Mucinhato R.M.D., Hakim M.P., Stedefeldt E., da Cunha D.T. (2022). What motivates consumer food safety perceptions and beliefs? A scoping review in BRICS countries. Foods.

[45] Nordhagen S., Lee J., Onuigbo-Chatta N., Okoruwa A., Monterrosa E., Lambertini E., Pelto G.H. (2022). “Sometimes you get good ones, and sometimes you get not-so-good ones”: Vendors’ and consumers’ strategies to identify and mitigate food safety risks in urban Nigeria. Foods.

[46] Teye E., Amuah C.L. (2022). Rice varietal integrity and adulteration fraud detection by chemometrical analysis of pocket-sized NIR spectra data. Appl. Food Res..

[47] Lee J., Pelto G.H., Nordhagen S. (2022). Beliefs, values and sociocultural patterns related to food safety in low- and middle-income countries: A synthesis of the descriptive ethnographic literature. Appetite.

[48] Rheinlander T., Olsen M., Bakang J.A., Takyi H., Konradsen F., Samuelsen H. (2008). Keeping up appearances: Perceptions of street food safety in Urban Kumasi, Ghana. J. Urban Health.

[49] Abate G.T., Bernard T., de Janvry A., Sadoulet E., Trachtman C. (2021). Introducing quality certification in staple food markets in Sub-Saharan Africa: Four conditions for successful implementation. Food Policy.

[50] Bene C., Heck S. (2005). Fish and food security in Africa. NAGA, WorldFish Center Quarterly.

[51] Igomu T. Using Chlorine Bleach in Fufu, Gari, Way to Kill People ‘Softly’—Experts. https://healthwise.punchng.com/using-chlorine-bleach-in-fufu-gari-way-to-kill-people-softly-experts/.

[52] Deudjui G., Chongwang J., Nakweya G. Poisons Used to Beautify Food in Africa. https://www.scidev.net/sub-saharan-africa/features/poisons-used-to-beautify-food-in-africa/.

[53] Idris A.B., Kebbi B. Stop Using Formalin, NAFDAC Warns Meat and Fish Sellers. The Guardian, 17 August. https://guardian.ng/news/stop-using-formalin-nafdac-warns-meat-fish-sellers/.

[54] Chilaka C.A., Mally A. (2020). Mycotoxin occurrence, exposure and health implications in infants and young children in Sub-Saharan Africa: A review. Foods.

[55] Ojuri O.T., Ezekiel C.N., Eskola M.K., Sarkanj B., Babalola A.D., Sulyok M., Hajšlová J., Elliott C.T., Krska R. (2019). Mycotoxin co-exposures in infants and young children consuming household- and industrially-processed complementary foods in Nigeria and risk management advice. Food Control.

[56] Ayeni K.I., Sulyok M., Krska R., Warth B., Ezekiel C.N. (2023). Mycotoxins in complementary foods consumed by infants and young children within the first 18 months of life. Food Control.

[57] Suman M., Cavanna D., Sammarco G., Lambertini F., Loffi C. (2021). Fighting food frauds exploiting chromatography-mass spectrometry technologies: Scenario comparison between solutions in scientific literature and real approaches in place in industrial facilities. Trends Analyt Chem..

[58] Liu R., Gao Z., Snell H.A., Ma H. (2020). Food safety concerns and consumer preferences for food safety attributes: Evidence from China. Food Control.

[59] Tran N., Shikuku K.M., Hoffman V., Lagerkvist C.J., Pincus L., Akintola S.L., Bailey C. (2022). Are consumers in developing countries willing to pay for aquaculture food safety certification? Evidence from a field experiment in Nigeria. Aquaculture.

[60] Liu R., Pieniak Z., Verbeke W. (2014). Food-related hazards in China: Consumers’ perceptions of risk and trust in information sources. Food Control.

[61] Soon J.M. (2020). Consumers’ awareness and trust toward food safety news on social media in Malaysia. J. Food Prot..

[62] Elliott C. Africa’s Food Fraud Problem Is Immense—But We Can Help. Queen’s Policy Engagement. http://qpol.qub.ac.uk/africas-food-fraud-problem-immense/.

[63] Teye E. Food Fraud in Africa: A Silent but Deadly Threat to Global Food Security. New Food. https://www.newfoodmagazine.com/article/159837/food-fraud-in-africa/.

